# In‐situ responses of temperate‐zone bats to climate change

**DOI:** 10.1111/nyas.15317

**Published:** 2025-03-20

**Authors:** Gerald Kerth, Janis M. Wolf

**Affiliations:** ^1^ Applied Zoology and Nature Conservation, Zoological Institute and Museum University of Greifswald Greifswald Germany

**Keywords:** adaptation, bat conservation, Chiroptera, demography, global warming, hibernation, phenotypic plasticity

## Abstract

There is growing evidence that human‐induced climate change poses a major threat to bats. As climate change progresses, we can only hope to mitigate its negative effects on bat populations by gaining a more comprehensive understanding of the complex interactions of all the factors involved. Drawing on recent evidence, largely from long‐term field studies of individually marked bats, we discuss the multiple impacts—positive and negative—of climate change on temperate heterothermic bats and their responses to climate change in situ. For example, there is increasing evidence that warmer summers and milder winters are leading to changes in the seasonal phenology of bats, which in turn may lead to species‐specific changes in demography, morphology, physiology, food availability, and roost use. We also highlight open research questions on the responses of bats to climate change. This includes better data on population trends and the underlying direct and indirect climate‐related causes for changes in mortality and reproductive success. In order to assess the long‐term impacts of climate change on bats, more information is needed about the relative importance of phenotypic plasticity and evolutionary adaptation in the responses of bats to climate change.

## Graphical abstract

There are multiple threats and responses of temperate bats to climate change at different stages of their annual life cycle. For example, different roost types may have advantages or disadvantages as climate change progresses. Bats show multiple responses to climate change, including changes in their seasonal phenology, demography, morphology, physiology, food availability, and roost use. The consequences for population persistence are likely to be species‐specific, but remain poorly understood.

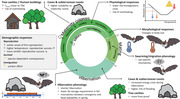

## INTRODUCTION

Bats are highly successful mammals from an evolutionary perspective. In the Eocene, the forests of the northern hemisphere were home to bats that were morphologically and behaviorally remarkably similar to the bats that live there today. Fossil records from the United States, France, and Germany revealed that bats have been able to fly and echolocate since at least 50 million years.^1^, ^2^ These features make bats unique among terrestrial mammals and allowed them to radiate into more than 1450 extant species.^1^

Earth's climate has changed dramatically several times since the Eocene^3^ and, as a result, the habitats of bats have also changed drastically over evolutionary time. Bats possess traits that enable them to cope with such long‐term environmental changes: notably, active flight, which facilitates range shifts, and facultative heterothermy in many species,^4^, ^5^ which allows them to better cope with adverse local conditions. Heterothermy describes the ability of endothermic animals to reduce their metabolism and, typically, body temperature to save energy.^4^ This energy‐saving metabolic state, known as torpor, allows heterothermic bats to better survive energetically stressful conditions than purely homeothermic mammals.^6^

Despite their extraordinary abilities, bats are generally considered vulnerable to rapid human‐caused environmental change, including climate change, with many species being endangered.^7^, ^8^, ^9^, ^10^ Like other animal taxa, bats theoretically can cope with climate change either locally (in situ^11^) or by colonizing more suitable locations (range shifts^12^, ^13^).

In this review, we focus on the in‐situ responses of bats to climate change—an area of research about which less is known compared to range shifts.^8^ Understanding how bats respond locally to changing conditions is critical for bat conservation in a world where dispersal and colonization of new areas is becoming increasingly difficult due to increasing habitat loss and fragmentation.^14^ In addition, females of most temperate bats are social and often philopatric to their natal colony, which may impede their ability to colonize new areas.^15^

As a result of the hidden life of most bat species, bat populations are often difficult to monitor and reliable data‐based population trends are very rare for bats, at least at regional and ecosystem levels.^9^, ^16^, ^17^, ^18^ This is particularly the case in the tropics, where there are fewer resources available for monitoring programs and many bat species are listed as data deficient by the IUCN.^9^ Although most bat species inhabit tropical regions,^19^ the majority of studies reporting large‐scale bat population trends come from temperate areas.^7^, ^20^, ^21^

Here, we focus on heterothermic bats that live in seasonal, temperate regions, mostly North America and Europe, where more data on population trends and in‐situ responses to climate change are available. Many of the data we discuss come from field studies on individually marked bats, including our own long‐term research on maternity colonies and the use of underground hibernacula in forest‐living bats in Germany. At the end of this review, we briefly summarize the current knowledge on the impacts of climate change on heterothermic bats and highlight some open research questions regarding their in‐situ responses to climate change.

## POTENTIAL IMPACTS OF CLIMATE CHANGE ON BATS

Threats of climate change may result from the direct effects of increasing weather anomalies, such as heat waves causing metabolic stress, as well as indirect effects, such as lower food availability during adverse weather.^10^, ^22^, ^23^, ^24^ Increasingly extreme weather events include severe storms, which can kill bats roosting in tree cavities when trees fall, or in flight, as has been suggested for the eastern red bat (*Lasiurus borealis*) during a severe thunderstorm over Lake Michigan.^25^ Secondary impacts of climate change such as changed human land use practices and human actions to reduce fossil fuel consumption, for example, installing wind turbines and insulating buildings, which all may threaten bats,^7^, ^9^ are outside the scope of this review.

In the following two sections, we provide an overview of the various associated direct and indirect threats from climate change to heterothermic temperate bats in terms of roosts and food availability, both of which are critical resources for bats.^10^ However, climate change could also have positive effects on bats, at least in temperate zones and in the short term,^26^, ^27^ which we will also discuss.

### Climate change and bat roosts

Most bat species spend the day in roosts that provide protection from weather and predators.^28^, ^29^ During specific stages of their reproductive cycle, some heterothermic bats prefer to maintain normothermia and thus benefit from warm roost temperatures, minimizing the energy required for thermoregulation.^30^ However, if roost temperatures become too hot, bats may experience higher metabolic costs^31^ and an increased risk of mortality.^32^, ^33^, ^34^ Moreover, males and females may differ in their thermal preferences due to their different lifestyles.^35^, ^36^, ^37^

Females and their offspring benefit from a stable and warm microclimate during the gestation and lactation period.^38^, ^39^, ^40^, ^41^ As a result, maternity colonies are often found in roosts with warm microclimates.^42^, ^43^, ^44^ Roost temperatures near the species‐specific thermoneutral zone (TNZ) enable bats to minimize their energy costs of thermoregulation without relying on torpor, which can adversely affect juvenile development.^30^, ^45^

Males may prefer colder roosts during the gestation and lactation periods of females, as they may benefit from entering torpor more often during the summer.^46^, ^47^, ^48^, ^49^ In late summer and autumn, before the onset of the mating season, this pattern may reverse, as males in some species prefer normothermia to promote spermatogenesis.^50^, ^51^ In contrast, females and juveniles may conserve energy by entering torpor for part of the day during this phase of their annual cycle.^38^, ^39^, ^40^, ^47^ Due to the critical importance of roosts for the survival and reproduction of bats,^28^ we take a closer look at the different roost types in light of climate change.

Underground sites, such as caves, unused mines, bunkers, and cellars, are typically more thermally stable than other types of bat roosts, with deep caves often reflecting the average ambient temperature.^52^, ^53^ In temperate regions, this means that underground sites are used by heterothermic bats for hibernation, but are often too cold for maternity colonies to successfully raise offspring.^54^, ^55^ Bat species that give birth and raise their offspring in caves in the warmer part of their range could, therefore, benefit from global warming during summer, as caves and other underground locations could become thermally more suitable for raising offspring even in colder parts of their distribution range (c.f. Ref. 56).

During hibernation, however, higher temperatures in underground hibernacula may lead to a faster depletion of fat‐reserves in hibernating bats (Refs. 13, 57, 58, but see Ref. 59 for the opposite effect). A further effect of warmer and wetter winters may be an increased risk of flooding in underground sites after sudden snow melts and/or strong rainfall events during the hibernating period.^60^, ^61^ These problems are aggravated by the fact that, in at least some bat species, individuals exhibit high fidelity to their specific hibernacula and show very little prospecting of alternative sites, limiting the likelihood that bats can easily move to new hibernation sites in case their original hibernaculum becomes unsuitable.^62^ Since most temperate‐zone bats hibernate in underground sites,^54^, ^63^ even species that roost in tree cavities or human buildings during the summer may be affected by higher and potentially more fluctuating temperatures and humidity in their hibernacula.

Roosts in house attics may overheat more quickly than caves during summer, yet are often used as roost for maternity colonies by species, such as greater mouse‐eared bats (*Myotis myotis*), which originally evolved to raise their offspring in the more stable conditions of caves.^64^ Moreover, unlike bats that naturally roost in tree cavities or rock crevices during summer, cave and attic roosting species are often not adapted to frequent roost switching.^65^ Thus, for species that exhibit high fidelity to their maternity roosts, attics or other human structures that are vulnerable to overheating could become ecological traps in a warming climate, unless there is a temperature gradient within the roost that the bats could use to avoid overheating.

The microclimate of crevices in rocks and human structures is probably often intermediate between that of caves and house attics, with less stable and possibly warmer temperatures than in caves, but cooler and possibly more stable temperatures than in most house attics.^66^, ^67^ Moreover, bat species that are adapted to roost in crevices also typically switch their roost more often than cave‐dwelling bats.^65^ Such roost‐switching behavior could make them less vulnerable to overheating than bats roosting in hot attics, if during heat waves roost temperatures rise gradually from day to day and the bats are able to respond by switching to cooler roosts in time.

Most temperate bats use tree cavities as summer roosts, but there are a few species that also hibernate in tree cavities. For example, two North American bat species, Rafinesque's big‐eared bat (*Corynorhinus rafinesquii*) and the southeastern myotis (*Myotis austroriparius*), roost in tree cavities in seasonally flooded swamp forests in both summer and winter. Although both species appear to be able to adapt their roost selection to the seasonal flooding risks, more severe flooding as a result of increased storm frequency and intensity due to climate change may become a threat to both species.^68^

Whereas tree cavities are generally less thermally stable than caves, they typically have a more stable microclimate than bat boxes.^69^, ^70^, ^71^ Forest‐dwelling bats have been found to show seasonal preferences for boxes versus tree cavities and are well known to consider the temperature of their roosts when deciding where to spend the day.^42^, ^72^, ^73^, ^74^ Since day and nighttime temperatures in roosts may not correlate well,^75^ roost temperatures at night—when bats select their roosts—may not reliably predict daytime temperatures. Choosing a cool roost at night could turn out to be a deadly trap the following day if this roost becomes hot under direct sun light. This risk has been demonstrated for sun‐exposed bat boxes,^32^, ^33^, ^34^ but may also occur in crevices of man‐made structures, such as house siding, or in natural roosts, for example, behind protruding bark in full sun.

In summary, climate change can have multiple and sometimes opposing effects on the suitability of bat roosts, depending on the type of roost, the season, and the bat species.

### Climate change and food availability

Outside the hibernation period, heterothermic bats are dependent on food availability for survival and reproduction.^54^, ^76^ Climate change can affect food availability for bats in a variety of ways. For example, annual insect biomass has been found to be positively correlated with summer temperature and spring precipitation in Germany.^77^ At the same time, more rainy nights may interfere with foraging efficiency and flight energetics in bats.^78^ Thus, changing rainfall patterns may lead to changed food availability for insectivorous bats.^10^ As insect activity is typically higher in warmer nights,^50^ insectivorous bats could benefit from warmer nights due to increased food availability. Depending on what bats optimize—maximum food intake or minimum time spent outside the roost to reduce predation risk—increased food availability could lead to either fewer, shorter—but more efficient foraging trips—or increased food intake and thus more energy available to maintain the body or invest in reproduction.

Species‐, sex‐, or age‐specific responses of bats to weather conditions could lead to phenological mismatches with their insect prey both during the critical period of emergence from hibernacula in spring and during the reproductive period in summer. During both periods, but particularly in spring, bats are highly dependent on food availability.^79^, ^80^ It is well‐documented that female bats adjust their breeding phenology to weather conditions in spring.^81^, ^82^, ^83^, ^84^, ^85^ If bats emerge from hibernation too early, with not yet enough food available, they may risk increased mortality.^86^ If females emerge too late, compared to the peak of food availability, they may lose valuable time and energy for optimal reproduction and, for example, start giving birth at times when seasonal arthropod availability has already begun to decline.^87^ Finally, it is also known that insects respond to climate change by changing their phenology.^88^ This can lead to further phenotypic mismatches if bats are unable to respond quickly enough in their own phenology to that of their prey.

Overall, the influence of climate change on food availability for bats is likely to be complex and dependent on the geographic region and ecosystem.

## BATS’ RESPONSES TO CLIMATE CHANGE

In the following sections, we report what is known about the behavioral, demographic, morphological, and physiological responses of heterothermic temperate‐zone bats to climate change.

### Behavioral responses

#### Phenology

The annual life cycle of heterothermic bats in temperate zones is closely linked to the bats’ seasonal habitat. Since almost all temperate bats are strict insectivores, there is very little food available to them in winter. Therefore, most temperate bats hibernate for several months during the cold season.^5^ Weather conditions can influence the timing of entry and emergence from hibernation in bats,^89^, ^90^ and there is growing evidence that bats shift the timing and duration of hibernation in response to changing seasonal weather conditions.^79^, ^86^, ^91^ Interestingly, species, sex, and age classes may respond differently, either shortening their hibernation in response to warmer fall and spring months, or showing the opposite response.^86^, ^89^, ^92^ While most of the observed responses appear to be due to individual plasticity, there is also some evidence of heritability in the hibernation phenology of two European bat species.^86^

#### Communal breeding and social behavior

The energetic benefits of social thermoregulation during the costly reproductive period is one of the likely reasons why, in most bat species, females form maternity colonies to communally raise their offspring.^93^ However, social thermoregulation cannot prevent overheating, and females and their offspring living in colonies that spend the day in warm roosts may be at increasing risk from heat stress as global warming continues. Finally, because warmer roost temperatures may reduce the need for social thermoregulation, global warming could lead to the formation of smaller roosting group sizes during the breeding season.^40^, ^43^. Interestingly, in parti‐colored bats (*Vespertilio murinus*), which is one of the few temperate bat species where males form separate colonies, males form larger aggregations on cold days to better profit from social thermoregulation during spermatogenesis.^50^

#### Mating

Very little is known about how climate change influences mating behavior in bats. In the temperate zones, mating is often very seasonal, typically peaking in late summer and fall,^94^, ^95^, ^96^ with some species also mating in spring.^97^ Because of their different temperature requirements during summer, males and females may respond differently to a changing climate in terms of the timing of their arrival at mating sites or fall migration. For example, in species in which mating occurs during autumn swarming at hibernacula (e.g., *Myotis bechsteinii*,^98^, ^99^ *M. nattereri*, *M. daubentonii*,^63^, ^95^ and *M. lucifugus* ^94^) or along their migration routes (e.g., *Pipistrellus nathusii*,^100^ *Nyctalus noctula*,^101^ *Lasionycteris noctivagans*,^102^ *Lasiurus cinereus*, and *L. borealis* ^103^), sex‐specific changes in seasonal phenology could result in a phenological mismatch between the sexes during the fall mating season.

#### Hibernation

It is known that bats choose their roosting locations within the hibernacula depending on the local microclimate and can thus avoid some of the fluctuations in ambient temperature.^104^, ^105^, ^106^ However, this behavior appears to be primarily an adaptation to cold climate rather than rising temperatures, as the interior of caves typically maintains a warmer and more stable microclimate than areas closer to the entrance.^106^ If bats decide to stay closer to the entrance of the hibernacula, they could experience colder temperatures on cold days, while they would face warmer temperatures during unusually warm periods in winter. Interestingly, there is evidence that bats adjust their fat reserves accumulated in the fall in response to warmer winters. A study on Schreiber's bent‑winged bat (*Miniopterus schreibersii*) showed that body condition at the onset and end of hibernation decreased over two decades, while the rate of mass loss also decreased.^59^ However, avoiding hibernation at all may not be a viable option for bats as long as insect availability is low in winter (Ref. 107, but see Ref. 108).

### Demographic responses

#### Reproduction

As with many animals, the breeding season of temperate bats is strongly linked to environmental conditions, with births usually coinciding with the time of year when food is most plentiful.^109^, ^110^ In most bat species, not all individuals within a colony reproduce every year, depending on their age but also on the prevailing weather conditions. Typically, 1‐year‐old females are less likely to reproduce than older females,^85^, ^111^, ^112^, ^113^ and conditions in spring generally influence reproductive rates in a given year.^81^, ^82^, ^114^ Female Bechstein's bats (*M. bechsteinii*) born in warmer summers have an increased probability of reproducing in their first adult year and, therefore, maintaining their lifetime reproductive success despite a shorter lifespan.^112^ Similarly, in greater horseshoe bats (*Rhinolophus ferrumequinum*), early‐breeding females had shorter life spans than late‐breeding females, while their lifetime reproductive success was not significantly different.^115^ Increasingly warmer summers due to climate change could, therefore, lead to shifts in reproductive success and thus have long‐term effects on population dynamics.

Temperature is not the only abiotic factor influencing reproductive traits; there is also evidence that rainfall has an impact on the reproductive success of bats.^10^, ^116^ As mentioned above, too little rain fall may negatively affect insect biomass,^77^ while heavy rainfall could hinder bats to forage successfully.^78^ However, the impact of precipitation patterns seems to be age‐ and/or species‐specific, depending on the foraging behavior of the given species. For example, in Natterer's bats (*Myotis nattereri*), increased precipitation during the lactation period reduces the likelihood that 1‐year‐old females reproduce, whereas there was no effect in females that were 2 years or older.^85^ In little brown bats (*Myotis lucifugus*) and the Yuma myotis (*M. yumanensis*), high levels of precipitation delayed parturition and reduced the proportion of reproductive females, suggesting that in wet years, some females skip parturition in order to maximize lifetime reproductive success.^117^ In greater horseshoe bat colonies in Britain, higher precipitation in spring had a negative impact on population growth, with a lag of 1 year, while higher spring temperatures had an immediate positive effect.^26^

#### Survival

Bats may experience species‐ and/or sex‐specific differences in survival during different seasons: some experience higher mortality in winter than in summer,^118^, ^119^, ^120^ others show the opposite pattern,^120^, ^121^ and some show no seasonal difference.^119^, ^122^ However, for most species, seasonal survival rates are unknown. If bats have lower mortality rates in winter because they are safer from predators while staying in their hibernacula, shorter hibernation periods may put them at higher risk because they spend more time in the riskier environment. The opposite could be true for species that have a higher mortality rate in winter than in summer. In order to assess the impact of climate change on bats, it is important to know whether bat populations are more winter or more summer regulated. Moreover, it has been shown for Bechstein's bats that rare catastrophic population crashes can drive population dynamics.^118^ Thus, increases in extreme weather events that cause elevated mortality could impact bat populations long before a gradual rise in average temperatures have an effect.

#### Immigration

Many temperate bat species show a high fidelity to their natal colony, making immigrations into maternity colonies a rare occurrence. In Bechstein's bats, this behavior is particularly pronounced, as females live in closed colonies with virtually no immigration.^123^, ^124^ Over more than 30 years of intensive monitoring of RFID‐tagged females, immigration has been documented only for two females that joined a colony in which they were not born.^124^, ^125^ This means that after population declines, colonies have to regrow based on their birth rates alone, and recovery can take a decade or more.^125^, ^126^ For example, the colony GB2 living in one of our study sites in southern Germany crashed in winter 2010/2011 from more than 40 adult females to just 17.^118^ It was only 10 years later, in 2021, that the number of females exceeded 40 again, and has since increased to over 60 adult females (G. Kerth, unpublished data). The original population crash was probably caused by the combination of an unusually cold fall followed by a cold spring. It is important to note that despite rising global temperatures and more frequent regional heat waves, unusual cold weather events in winter will still occur and may even be linked to the overall climate change pattern.^127^ Although the frequency of these cold events may decrease over the coming decades, those that do occur could bring even more severe consequences.^128^

### Physiological responses

While the ability to fly allowed bats to exploit novel ecological niches, it also sets strict limits on their body size and mass.^129^ As a result, the vast majority of bat species have a body mass below 30 g (median: 12 g), with only very few species of flying foxes exceeding 1 kg.^130^ This limitation of low body mass and relatively small body size has significant physiological impacts on bats, influencing processes such as thermoregulation and reproduction. With rising average temperatures and more frequent extreme weather events, temperate‐zone bats may thus encounter considerable physiological challenges.^8^, ^10^

Bats in temperate regions are exposed to strong seasonal and daily fluctuating weather conditions and feed primarily on seasonally available food sources such as insects.^131^ Consequently, temperate‐zone bats spend much of their annual cycle in ambient temperatures below their TNZ, conditions that require substantial energy for thermoregulation to maintain normothermia.^132^ During the winter months, temperate bats cannot compensate for this higher energy requirement by increasing food intake, as prey is scarce during this time. Thus, they typically enter hibernation for several months, during which they significantly reduce their energy consumption.

Having already discussed the potential impacts of climate change on hibernating bats and their behavioral responses to changing winter conditions in the section on hibernation behavior, we now focus on how climate change may affect the physiology of temperate‐zone bats during the active seasons. Given the fact that the TNZ of small endotherms is located at relatively high temperatures,^133^ an increase in average temperatures is likely to cause ambient temperatures for many temperate species to approach the lower limit of their TNZ, theoretically reducing their energy requirements for thermoregulation.^134^, ^135^ However, the extent to which this will actually be effective is unclear as many bat species have adaptations to energetically buffer cold or unfavorable conditions. For instance, in species that form maternity colonies, social thermoregulation—where individuals huddle together to reduce heat loss—is a common behavior to conserve energy while roosting during cold days.^93^, ^136^, ^137^

In even colder temperatures or when foraging is temporarily not possible due to adverse weather conditions such as high precipitation or wind speeds, most temperate bats are able to also use torpor to save energy during the summer.^5^ However, using torpor extensively during the prelactation and lactation periods is associated with prolonged gestation times^138^, ^139^ and reduced milk‐production.^45^ While rising average temperatures could reduce the need for social thermoregulation or torpor to cope with cold spells, at least torpor is still likely to be useful for enduring the more frequent extreme weather events associated with climate change.^6^

By reducing thermoregulatory costs, warmer temperatures may also alter the energy budgets of mothers available for reproduction. A field study on Bechstein's bats found that mothers roosting in bat boxes artificially heated to the TNZ during the lactation period did not have better body condition (calculated as the scaled mass index^140^) after weaning than mothers in boxes that were not heated.^141^ This suggests that the energy savings resulting from less costly thermoregulation in heated roosts are allocated directly into other metabolic processes during this energy‐demanding period. Likely, the investment is channeled into the current offspring, which is consistent with the observation of larger body sizes attained by juveniles that have grown up in heated roosts (see section about morphological responses).^142^

While a slight increase in average ambient temperature could be energetically beneficial, excessive heat waves and droughts can have dire consequences for temperate‐zone bats—overheating can cause metabolic stress^31^ and ultimately lead to death.^143^ Recent mass mortalities of bats due to extreme heat have been reported primarily from tropical and subtropical regions, particularly among relatively large species of flying foxes that roost in trees exposed to the open sky.^144^, ^145^, ^146^, ^147^ Even if such observations are fortunately not yet frequent in the temperate zones, there is also concern that an increase in extremely hot days associated with climate change could pose a threat to bats living there.^148^ Different species may have a higher or lower heat tolerance depending on their preferred roost type. For example, foliage‐roosting hoary bats (*L. cinereus*) show higher tolerance to heat than little brown bats (*M. lucifugus*) and silver‐haired bats (*L. noctivagans*), both of which typically roost in more temperature‐buffered roosts in tree cavities.^149^ Of particular concern are suboptimally placed bat boxes, which are often provided as a compensatory measure for the destruction of natural roosting opportunities. Bat boxes exposed to direct sunlight are prone to overheating and thus could lead to thermal stress with potentially fatal consequences.^32^, ^33^, ^150^

Originally, heterothermy was predominantly seen as an adaptation to cold temperatures or to low food availability, but there is evidence that tropical bats can combine torpor with facultative hyperthermia at hot temperatures to save energy while roosting.^151^ However, it is not yet known whether bats in temperate zones can also make use of such hot torpor. A study on common noctule bats (*N. noctula*) has shown that torpor induced at cool temperatures can lead to energy savings even when ambient temperature rises to as high as 30°C.^152^ However, it remains unclear whether torpor can also be induced at high temperatures, or what happens at temperatures above the TNZ.^152^

### Morphological responses

In addition to changes in phenology, changes in morphology are the best documented in‐situ responses to climate change, both in animals in general and in bats.^153^, ^154^, ^155^ For many animal species, there is evidence that individuals become smaller as the climate gets warmer, as would be expected according to Bergmann's rule.^156^ However, this phenomenon is not universal; some species show no changes in body size, while others actually become larger in a warmer environment.^153^ Such differences in species‐specific responses have also been observed in bats.^27^, ^83^, ^157^, ^158^, ^159^, ^160^, ^161^

Overall, evidence on whether and how rising environmental temperatures affect body sizes in bats is mixed and the underlying causes are often unclear or not directly related to global warming. Cranial size of Kuhl's pipistrelle (*Pipistrellus kuhlii*) has been increasing over the last century, but this is more likely related to the effects of urbanization and associated changes in resource availability.^162^ In horseshoe bats in Italy, *R. ferrumequinum* showed an increase in body size during the 20th century,^161^ whereas *R. hipposideros* did not, although a positive correlation between body size and latitude was observed.^160^ In China, *Hipposideros armiger* has increased in size over recent decades, a change linked to shifting food availability due to land use changes, rather than as a direct result of a warmer environment.^163^ Data from another bat species found in China, *Rhinolophus pusillus*, suggest a decrease in body size with increasing winter temperatures, supporting the heat conservation hypothesis often linked to Bergmann's rule.^164^ Among 20 bat species studied in North America, a correlation between increasing body mass and average annual temperature was observed in only a few species.^157^

An increase in body size in animals is typically explained by greater food availability, allowing juveniles to grow larger.^165^ For example, little brown bats decreased in body size as insect abundance or availability decreased in years with more days of precipitation during the summer months.^166^ In contrast, heating experiments in two wild Bechstein's bats colonies provide evidence that warmer roost temperatures can directly lead to larger forearm length in juvenile bats of both sexes.^142^ The size of this effect is illustrated by the finding that in this sexually dimorphic species, male juveniles reared in optimally warm roosts reached forearm lengths comparable to those of juvenile females reared in colder and more variable roost temperatures. This field experiment provides an explanation for previous observations that warmer summers lead to larger female Bechstein's bats.^83^

By analyzing the heritability of forearm length in differently warm environments, Mundinger et al. showed that this increase in body size is largely due to phenotypic plasticity.^167^ Daughters born in summers that were warmer than the summers in which their mothers were born grew larger than their mothers. If daughters were born in colder summers than their mothers, these daughters often did not reach the size of their mothers. Overall, the heritability of forearm length was lower in hot summers compared to average and cold summers, suggesting a strong influence of summer temperature on body size via phenotypic plasticity.

## SYNTHESIS AND OUTLOOK

Anthropogenic climate change is only one of many current threats to bats. Habitat loss and habitat fragmentation, persecution and exploitation, pollution and pesticides, introduction of invasive species, and diseases all contribute to the decline of bat populations around the world.^7^, ^9^ Unfortunately, interactions and positive feedback loops between the various threats to bats may be worsening the situation for population persistence in a rapidly changing world. As a global process, climate change can increase the impacts of other anthropogenic threats on animal populations, for example, by facilitating the spread of invasive species and increasing habitat loss.^168^ For example, in the highly endangered New Zealand long‐tailed bat (*Chalinolobus tuberculatus*), climate warming could lead to an increased predation risk by introduced rats and stoats.^169^ The spread of invasive plants, which may be facilitated by climate change, may also pose a threat to bats if they degrade the quality of bat habitats (c.f. Ref. 170).

In our review, we discuss the diverse impacts of climate change on heterothermic bats of the temperate zones. For example, increasing evidence suggests that warmer summers and milder winters can shift the seasonal phenology of bats, including an earlier start to the breeding season and altered hibernation periods. In turn, these shifts in combination with changes in ambient temperature and other environmental factors may affect food availability and the bats’ roosting and foraging behavior, physiology, morphology, and demography.

Shifts in the thermoregulatory requirements of temperate bats due to a warming environment have been linked to morphological and physiological consequences that may have species‐specific implications for population dynamics. In Bechstein's bats, increasingly warmer summers lead to juveniles reaching larger body sizes,^83^ which in turn is associated with higher mortality rates and shorter life spans.^112^, ^118^ On the contrary, in Natterer's bats, larger females have a higher survival rate, and in this species, at least populations that currently live in colder regions could profit from warmer summers.^27^ Larger female Bechstein's bats appear to be able to maintain their lifetime reproductive success despite a shorter life span due to an accelerated pace of life.^112^ However, this strategy may prove risky and unsustainable in the long term if climate change results in more years that are unfavorable for reproduction or if insects continue to decline due to intense human land use or climate change.^77^, ^171^ Overall, we are only starting to understand the complex interactions between all these factors. Thus, compared to many of the other human‐caused threats to bats, still relatively little is known about how climate change is affecting bat populations (see also Refs. 8, 10, and 61).

To better understand the complex and sometimes opposing effects of climate change on bats, we need more and better monitoring data on population trends and a better understanding of the causes of changes in mortality and reproductive success. To date, detailed investigations of demographical, morphological, and physiological responses—and their interactions—have been conducted for only a few bat species. Expanding this research to include a wider range of species would significantly enhance our understanding of these critical responses to climate change. To be able to predict the long‐term impacts of climate change on bats, we also need to know more about the relative importance of phenotypic plasticity and evolutionary adaptation in the responses of bats to climate change. As climate change continues to progress, we can only hope to mitigate its negative impacts for bat populations by gaining a more comprehensive understanding of the complex interactions of all the factors involved.

## AUTHOR CONTRIBUTIONS

G.K. and J.M.W. wrote this article together.

## COMPETING INTERESTS

The authors declare no competing interests.

### PEER REVIEW

The peer review history for this article is available at: https://publons.com/publon/10.1111/nyas.15317

## Data Availability

The data that support the findings of this study are available on request from the corresponding author. The data are not publicly available due to privacy or ethical restrictions.
